# Intra- and Inter-clade Cross-reactivity by HIV-1 Gag Specific T-Cells Reveals Exclusive and Commonly Targeted Regions: Implications for Current Vaccine Trials

**DOI:** 10.1371/journal.pone.0026096

**Published:** 2011-10-12

**Authors:** Lycias Zembe, Wendy A. Burgers, Heather B. Jaspan, Linda-Gail Bekker, Helba Bredell, Gwynneth Stevens, Jill Gilmour, Josephine H. Cox, Patricia Fast, Peter Hayes, Eftyhia Vardas, Carolyn Williamson, Clive M. Gray

**Affiliations:** 1 Division of Medical Virology, Institute of Infectious Disease and Molecular Medicine, University of Cape Town, Cape Town, South Africa; 2 Division of Immunology, Institute of Infectious Disease and Molecular Medicine, University of Cape Town, Cape Town, South Africa; 3 The Desmond Tutu HIV Centre, Cape Town, South Africa; 4 International AIDS Vaccine Initiative, New York, New York, United States of America; 5 Department of Medical Virology, University of Stellenbosch, Stellenbosch, South Africa; 6 National Institute for Communicable Diseases, Johannesburg, South Africa; University of Toronto, Canada

## Abstract

The genetic diversity of HIV-1 across the globe is a major challenge for developing an HIV vaccine. To facilitate immunogen design, it is important to characterize clusters of commonly targeted T-cell epitopes across different HIV clades. To address this, we examined 39 HIV-1 clade C infected individuals for IFN-γ Gag-specific T-cell responses using five sets of overlapping peptides, two sets matching clade C vaccine candidates derived from strains from South Africa and China, and three peptide sets corresponding to consensus clades A, B, and D sequences. The magnitude and breadth of T-cell responses against the two clade C peptide sets did not differ, however clade C peptides were preferentially recognized compared to the other peptide sets. A total of 84 peptides were recognized, of which 19 were exclusively from clade C, 8 exclusively from clade B, one peptide each from A and D and 17 were commonly recognized by clade A, B, C and D. The entropy of the exclusively recognized peptides was significantly higher than that of commonly recognized peptides (p = 0.0128) and the median peptide processing scores were significantly higher for the peptide variants recognized versus those not recognized (p = 0.0001). Consistent with these results, the predicted Major Histocompatibility Complex Class I IC_50_ values were significantly lower for the recognized peptide variants compared to those not recognized in the ELISPOT assay (p<0.0001), suggesting that peptide variation between clades, resulting in lack of cross-clade recognition, has been shaped by host immune selection pressure. Overall, our study shows that clade C infected individuals recognize clade C peptides with greater frequency and higher magnitude than other clades, and that a selection of highly conserved epitope regions within Gag are commonly recognized and give rise to cross-clade reactivities.

## Introduction

The development of a safe, globally effective and affordable vaccine offers the best hope for the future control of the HIV pandemic. One of the major challenges in developing such a vaccine is the high degree of genetic diversity the virus exhibits. The extensive genetic variation of HIV is fuelled by high mutation, recombination and replication rates, partly driven by host cellular and humoral immune pressure [1], [2]. As there is a need to test vaccines in clinical trials quickly and efficiently, where candidate vaccines may have been designed for one clade and be tested in populations where a different clade predominates, the ability to predict cross-clade epitope coverage is important.

T-cell immunity has been found to play a role in HIV control [3]–[5]. The importance of responses to Gag is well documented, with studies showing that the magnitude of anti-Gag CD8+ T-cell responses inversely correlates with plasma viral load, [6], [7] and that preferential targeting of this protein during infection leads to lower viral load [6], [8], [9]. Other studies have shown that the breadth of anti-Gag T-cell responses is associated with lower viral loads [7], [10] and collectively, these data strongly implicate Gag as an important target of HIV-specific T-cells for inclusion in candidate preventative vaccines. Of major importance for preventative vaccine development is the identity of regions within the HIV-1 proteome that can be targeted by T-cells and that are cross-reactive between different viral clades. As several HIV-1 vaccine candidates are at different stages of development, it is important to predict whether vaccines based on one clade may be effective in regions where different clades circulate. Previous studies that examined cross-clade HIV-1 Gag T-cell immune responses in an environment of multiple circulating clades [11]–[13] have found that HIV-infected individuals can mount robust cross-clade HIV-specific T-cell immune responses, but with a preference for the predominant circulating or infecting clade [14], [15].

South Africa has a high incidence of HIV-1 and is dominated by clade C, and a number of phase I and II trials and one phase IIb efficacy trial have taken place there, testing constructs based on clade A, B and C-based candidate vaccines [16]–[18]. Following on from the first demonstration of vaccine-induced protection from HIV-1 aquisition in the RV144 trial in Thailand [19], follow-up trials in high incidence settings such as South Africa are currently being planned. It is likely that both clade C and non-C based immunogens will be tested there in the future, and the ability to predict the level of T-cell coverage and cross-reactivity is thus important. In this study, we examined intra- and inter-clade cross-reactivity of HIV-1-specific T-cell responses to Gag, using peptides matching candidate South African and Chinese clade C vaccine contructs and compared these with clades A, B and D consensus-based peptides. The South African and Chinese *gag* genes have been included in candidate HIV-1 vaccines that have been tested for safety in phase I clinical trials [20]–[22]. We performed this study using a South African clade C-infected population, where we sequenced the infecting virus from each individual and assessed T-cell responses to the different peptide sets. This allowed us to explore two inter-related aims: a) to identify the location of commonly and exclusively targeted epitope regions in Gag and relate these to the level of virus variability; b) to identify the extent of intra- and inter-clade recognition using peptide sets that match vaccine inserts.

## Results

### Participant characteristics

Immunological data were available for 39 participants. The median age was 28 years (range 22–47 years). The median CD4 count was 492 cells/µl (range 295–1437 cells/µl; Table 1). Thirty-six participants had a response to at least one Gag peptide from one of the five peptide sets used. Seventeen participants who had Human Leukocyte Antigen (HLA) A and B typing data available (Table S1) had their reactive peptides further characterized.

**Table 1:** Summary of clinical data of study participants.

Characteristic	Median (Range)				
Age (Years)	28 (22–47)				
Plasma HIV RNA (Copies/ml)	11000 (200−260000)				
CD4 Count (cells/µl)	492 (295–1437)				
Frequency of HIV-specific T-cell responses	C_Du422_	C_CH_	B	A	D
N (%)	36 (92)	35 (90)	27 (69)	30 (77)	34 (87)

N (number of participants)  =  40. N = 39 participants were screened for immunological responses and 36 had responses to at least one Gag peptide. CDu422: South African clade C, CCH: Chinese clade C; A, B and D are clades A, B, and D sequences.

### Genetic distances and epitope coverage between the infecting virus sequence and peptide sets

**Figure 1:**
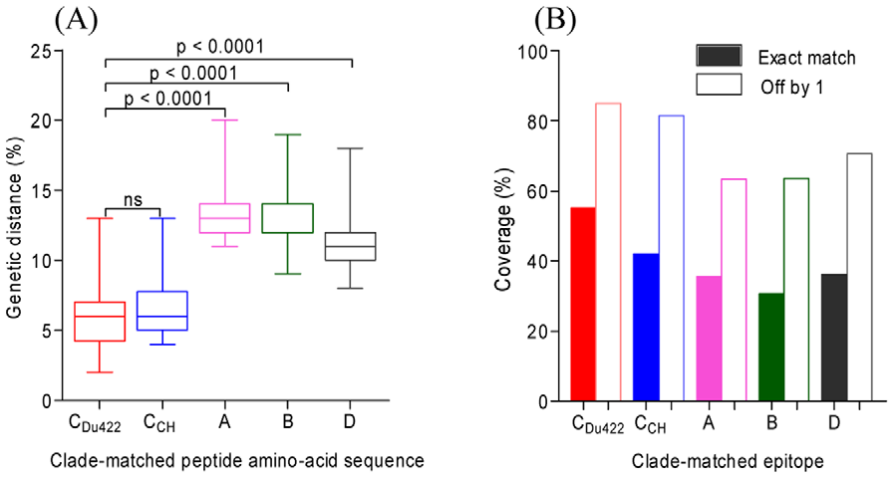
The relationship between infecting viral sequences and peptide amino-acid sequences. (A) Amino acid distances between infecting viral sequences and ELISpot peptide reagent sequences. The median amino acid distance for each peptide set is represented by a solid line. Differences were tested for significance by one way ANOVA test and Dunn's test. (B) Predicted 9mer epitope coverage of two current candidate vaccine peptide sequences (C_Du422_ and C_CH_) and consensus clades A, B, and D peptide sequences. For each peptide set, the values shown in a solid bar are exact matches while the open bars show coverage when mismatched by 1 amino acid. Analysis was performed using the p17p24p2 Gag peptides from 39 participants.

We first determined the genetic distance between the peptide reagents used in the ELISPOT assays and the infecting viral sequences from each of the participants. All study subjects were confirmed as being infected with HIV-1 clade C. Genetic distances for the South African clade C_Du422_, Chinese clade C, a synthetic B/C recombinant (C_CH_) and clade B peptide sets were based on the full length Gag amino acid sequences, whilst those for the other clades were based only on the p17p24p2 regions. The C_Du422_ and C_CH_ peptide sequences were equally genetically similar to the infecting viral sequences of the participants studied, with a median amino acid distance for C_Du422_ of 5.5%, and for C_CH_ of 6% (range, 4–15%; Figure 1A). However, the genetic distance between the peptide sets matching consensus clades B, A and D with the infecting clade C sequence was significantly greater (p<0.0001), with median distances of 12%, 13% and 11%, respectively (Figure 1A).

We next investigated differences in antigenic potential of the sequences, by examining the proportion of matching T-cell epitope-length peptides within the infecting viral sequences, compared to the peptide sets being tested. The coverage of putative 9mer epitopes within infecting sequences was similar for C_Du422_ and C_CH_ (55% and 53% respectively, Figure 1B). Consistent with the increased amino acid divergence in peptide sets A, B and D from the infecting virus (Figure 1A), matched epitope coverage was lower for these peptide sets, at 33%, 30% and 37%, respectively (Figure 1B). When 9mers were aligned that differed by one amino acid, there was a similar trend and an increased frequency of epitope coverage, at 85%, 81%, 63%, 64% and 71% for peptide sets C_Du422_, C_CH_, A, B and D, respectively (Figure 1B). These data suggest that intra-clade T-cell reactivity may be similar due to lower genetic divergence compared to between clades.

### Preferential recognition of clade C peptides

**Figure 2:**
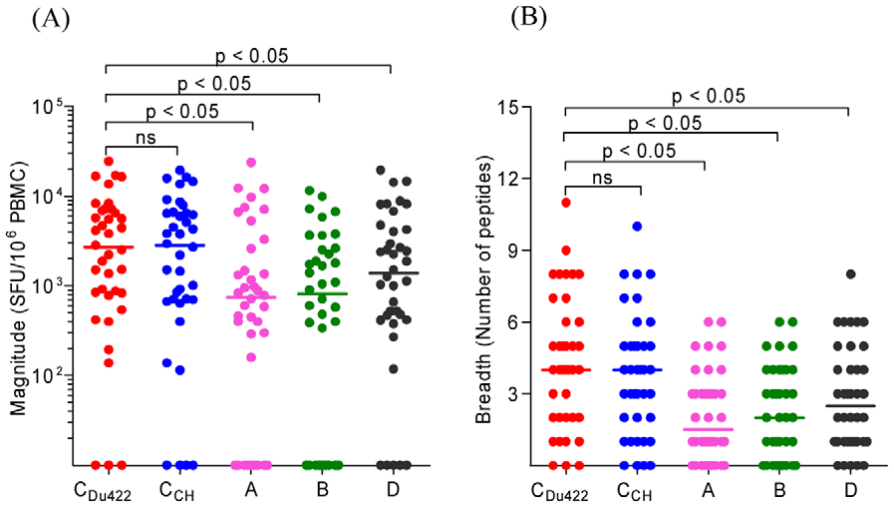
Recognition of HIV-1 Gag peptides from clades A, B, C_Du422_, C_CH_ and D sequences in HIV-1 clade C infected individuals from South Africa. (A) Total magnitude of Gag-specific T-cells (SFU/10^6^ PBMC) against individual peptides for each study individual. Values are shown in log scale. (B) The minimum number of responses per peptide set for each study individual. Data is based on the p17p24p2 region of the Gag protein. Significant differences were tested for using one way ANOVA followed by a Dunn's post-test.

At the first level of cross-clade analysis, we wished to identify whether the magnitude of ELISPOT responses was equally distributed between the different peptide sets. All comparisons between peptide sets were based on the p17p24p2 region of the Gag protein. For the C_Du422_ and C_CH_ sets, there was no difference between the median magnitude of response expressed as SFU/10^6^ PBMC, at 2690 (range 0 to 24550) for C_Du422_ and 2828 (range 0 to 19407) for C_CH_ (p>0.05; Figure 2A). Conversely, the magnitude of responses to other clades was significantly lower, at 750, 810 and 1390 for clades A, B and D, respectively (p<0.05; Figure 2A). A similar trend was observed for the breadth of responses, with a larger median number of reactive peptides being recognized in the two clade C based peptide reagents, C_Du422_: 4 (range 1–11) and C_CH_: 4 (range 0–10) when compared to clades A: 1 (range 0–6), B: 2 (range 0–6) and D: 3 (range 0–8) peptide sets (Figure 2B). Collectively, these data show that peptides more closely matched to the infecting autologous sequence result in higher magnitude responses and wider breadth of coverage, consistent with the predicted epitope coverage shown in Figure 1B.

### Peptide variability and cross-reactivity

**Figure 3:**
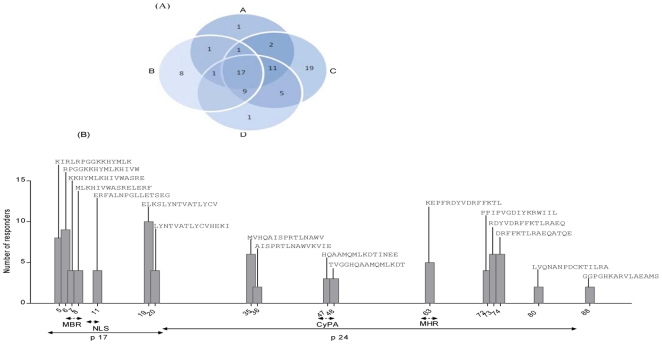
Characterisation of HIV-1-specific T-cells recognizing the five peptides sets investigated in the study. (A) The contribution of each peptide variant to the overall HIV-1-specific T-cell responses in the study. Shown in each subset of the Venn diagram is the number of peptides recognized in each and overlapping between the four HIV-1 clades. Reactive peptides from all study individuals were combined after removing overlapping peptides. Each peptide was exclusively assigned to a recognition category depending on the clade in which it was identified. If a peptide was recognized in more than one category, the category that gave the highest cross-reactivity was considered. The two clade C peptide sets (C_Du422_ and C_CH_) were combined. (B) HIV-1 Gag cross-reactive epitope hotspots. The Gag region in which the peptides are located is shown, MBR: membrane binding region; NLS: nuclear localization signal; CyPA: Cyclophilin A binding region; and MHR: major homology region.

The second level of analysis consisted of identifying the numbers of mutually (cross-reactive) and exclusively recognized (recognition by one clade only) peptides within the five peptide sets tested for reactivity. A total of 84 peptides were recognized by the study participants, with 29 peptides being exclusively recognized in one clade only. Nineteen peptides were exclusive to clade C (either C_Du422_ or C_CH_), 8 to clade B, and one peptide each to clades A and D peptide sets (Figure 3A). Between the two clade C peptide sets, 6/19 peptides were recognized exclusively in the C_Du422_ peptide set and 13/19 peptides were common between the two peptide sets. There were seventeen peptides that were mutually recognized across all four clades. Of these, 7 were positioned in p17 and 10 within the p24 region of the Gag protein (Figure 3B). The remainder of the peptides were recognised in two or three of the clades.

**Figure 4:**
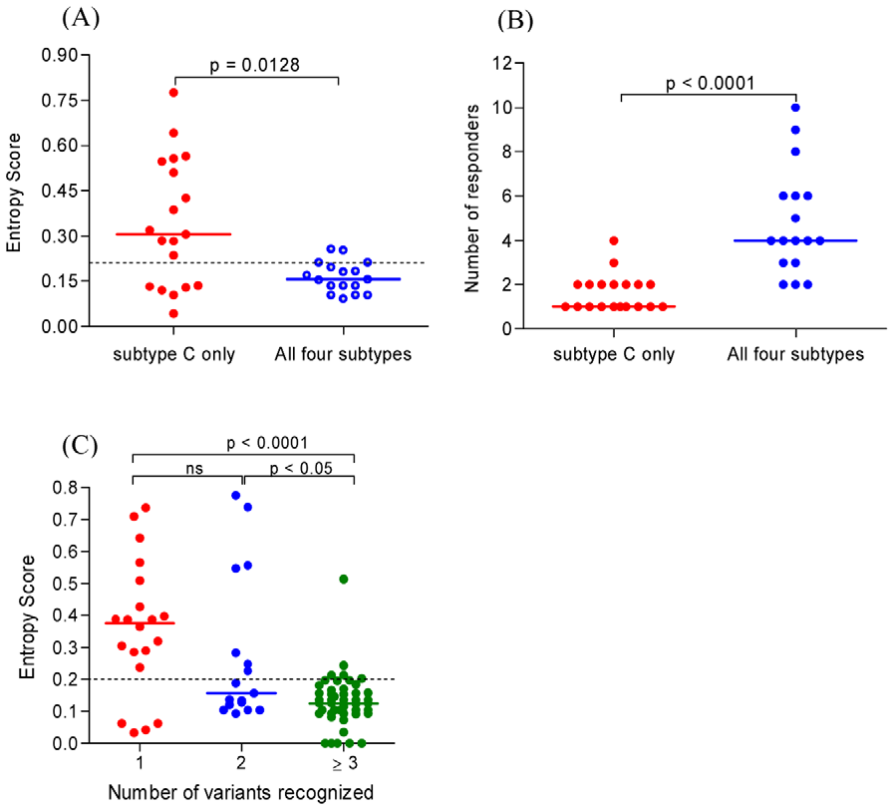
Characterization of mutually and exclusively recognized peptides. (A) Comparison of entropy scores between peptides recognized exclusively by clade C peptide reagents (red) and those mutually recognized in all the four clades tested (purple). (B) Frequency of responders to epitopes recognized by clade C reagents (red) compared to those mutually recognized in all four clades (purple). (C) Comparison of entropy score of peptides with different levels of cross-reactivities. Peptides were categorized into those recognized in one (red), two (purple) or three or more (green) variants of the peptide sets. The non-parametric one way ANOVA and Kruskal-Wallis test was used to test for differences in the median entropy scores among the different recognition categories.

In an attempt to understand the basis of mutual or exclusive recognition of peptides, we compared the Shannon entropy score for the 17 peptides cross-recognized and the 19 peptides exclusively recognized from the clade C peptide sets (Figure 4A). It was evident that peptides exclusively recognized had significantly higher entropy than the mutually recognized peptides (p = 0.0128). The bulk of these high entropy peptides were also the least recognized within the cohort (Figure 4B). These data suggest that exclusive recognition of peptides is related to clade C-specific variability within the epitopes. Not all peptides exhibited this pattern of low entropy and mutual recognition or high entropy and exclusive recognition, and Table 2 shows a representative example of an individual in the cohort (CC23) recognizing 11 peptides, in some cases despite extensive amino acid variability. Peptides 63 and 80 are examples of mutually recognized peptides regardless of amino acid change D→E and T→S within the each of the peptides, respectively (Table 2). These variations are most likely tolerated as they fall outside HLA anchor motifs or T-cell receptor (TCR) contact residues. In contrast, peptides 7, 15 and 32 were exclusively recognized due to variation in residues important for recognition, and hence not cross-reactive (Table 2). Peptides that were reactive despite differences from the infecting viral sequence may have had variations in ‘tolerated’ residues for peptide binding and conformation, or the epitopes may indeed have been presented in infected persons as a result of minor viral variants that we did not detect by population sequencing of the dominant virus.

**Table 2:** Characterization of reactive peptides recognized in study individual CC 23.

Peptide number	Name	Sequence	Restricting HLA	SFU/10^6^ PBMC
a7	Autologous	GKKRYMLKHIVWASRE	A*0201	
	CDu422	KKHYMLKHIVWASRE		1750
	CCH	KKHYMLKH*L*VWASRE		940
	B	*G*KK*K* Y*K*LKHIVWASR		0
	A	KK*K* Y*R*LKH*L*VWASRE		0
	D	KK*K* Y*R*LKH*L*VWASRE		0
a11	Autologous	EKFALNPGLLETSDG	A*0201	
	CDu422	ERFALNPGLLETSEG		130
	CCH	ERFALNPGLLETSEG		130
	B	*L*ERFA*V*NPGLLETSE		0
	A	ERFALNP*S*LLET *A*EG		0
	D	ERFALNPGLLETSEG		130
15	Autologous	KQIIKQLQPALQTGT	A*02	
	CDu422	KQIMKQLQPALQTGT		0
	CCH	KQI*I*KQLQPALQTGT		0
	B	C*R*QI*LG* QLQP*S*LQTG		120
	A	*CQ*QIM*E* QLQ*S*AL*K*T*S* *E*		0
	D	KQI*IG* QLQPA*I*QTG*S*		0
19	Autologous	EELKSLFNTVATLYCV	B*0801	
	CDu422	ELKSLYNTVATLYCV		0
	CCH	EL*R*SL*F*NTVATLYCV		1280
	B	EEL*R*SLYNTVATLYC		520
	A	LKSL*F*NTVATLYCVH		2353
	D	EL*R*SLYNTVATLYCV		480
b32	Autologous	SQVSQNYPIVQNLQGQMV	Unknown	
	CDu422	SQNYPIVQNLQGQMV		0
	CCH	SQNYPIVQNLQGQMV		0
	B	SQNYPIVQNLQGQMV		0
	A	*KV*SQNY*ALKHRAYEL*		0
	D	*SQV*SQNYPIVQNLQG		160
38	Autologous	NAWVKVIEEKAFSPEI	B*4501	
	CDu422	AWVKVIEEKAFSPEV		470
	CCH	AWVKVIEEKAFSPEV		470
	B	*N*AWVKV*V* EEKAFSPE		0
	A	AWVKVIEEKAFSPEV		470
	D	AWVKVIEEKAFSPEV		470
59	Autologous	AGTTSTLQEQIAWMTS	A*0201	
	CDu422	GTTSTLQEQIAWMTS		150
	CCH	GTTSTLQ*G*QIAWMTS		0
	B	*A*GTTSTLQEQI*G*WMT		0
	A	GTTSTLQEQI*G*WMTS		0
	D	GTTSTLQEQIAWMTS		150
63	Autologous	NPPIPVGEIYKRWIIL	B*0801	
	CDu422	PPIPVGDIYKRWIIL		6670
	CCH	PP*V*PVG*E* IYKRWIIL		5980
	B	*N*PPIPVG*E* IYKRWII		5320
	A	PPIPVGDIYKRWIIL		6670
	D	PPIPVG*E* IYKRWIIL		5520
80	Autologous	LLTQNANPDCKTILRA	B*0801	
	CDu422	LVQNANPDCKTILRA		7470
	CCH	LVQNANPDCKTILRA		7470
	B	*L*LVQNANPDCKTIL *K*		3920
	A	LVQNANPDCK*S*ILRA		2653
	D	LVQNANPDCKTIL *K*A		1240
89	Autologous	GHKARVLAEAMSQVGH	A*0201	
	CDu422	HKARVLAEAMSQTNS		110
	CCH	HKARVLAEAMSQ*A*N*G*		0
	B	*G*HKARVLAEAMSQ*V* *T*		0
	A	HKARVL*GTGARASV* *L*		0
	D	HKARVLAEAMSQ*A*TN		200
107	Autologous	FLGKIWPSHKGRPGN	A*0201	
	CDu422	FLGKIWPSHKGRPGN		450
	CCH	FLGKIWPSHKGRPGN		450
	B	FLGKIWPSHKGRPGN		450
	A	Not available^c^		N/A
	D	Not available		N/A

a The previously described HLA allele to restrict the epitope was absent in the individual, however HLA A*0201 was found to be a strong binder to the epitope within this peptide with a binding affinity of 5 nM for peptide 7 and a weak binder with an affinity of 135 nM for peptide 11, using NetMHC.

b No epitope described nor predicted to bind to HLA alleles of this participant in this peptide. ^c^Clades A and D had no p15 region of the Gag protein.

Some peptide variants with substitutions in regions flanking the epitope showed discordant recognition patterns regardless of matching epitope sequence, possibly due to additional non hydrophobic amino acids at the C-terminal or N-terminal that are not well tolerated by class I alleles [63].

ELISPOT reactivity is shown for variants that were reactive, while 0 denotes those that were not reactive. Italicized letters in the peptide sequence indicate amino acid mismatches between that peptide variant and the C_Du422_ sequence. The bold letters show amino acid mismatches between the peptide variant sequence and the infecting virus. The predicted epitope in each variant is underlined. SFU: Spot Forming Units (per 10^6^ PBMC).

Having observed that mutual or exclusive recognition of variant peptides was dependent on entropy; we extended the analysis to all 84 peptides in the study, and categorized them into those that were recognized in 1, 2 or≥3 of the peptide variants. Consistent with the previous data, exclusive peptide recognition was characterized by significantly higher entropy when compared to recognition in 2 or≥3 clades (p<0.05 and p<0.0001, respectively; Figure 4C). This resulted in a significant negative correlation between the degree of recognition and peptide entropy (r = −0.37, p = 0.0005).

### The Impact of Host HLA and Epitope Recognition by HIV-1-specific T-cells

**Figure 5:**
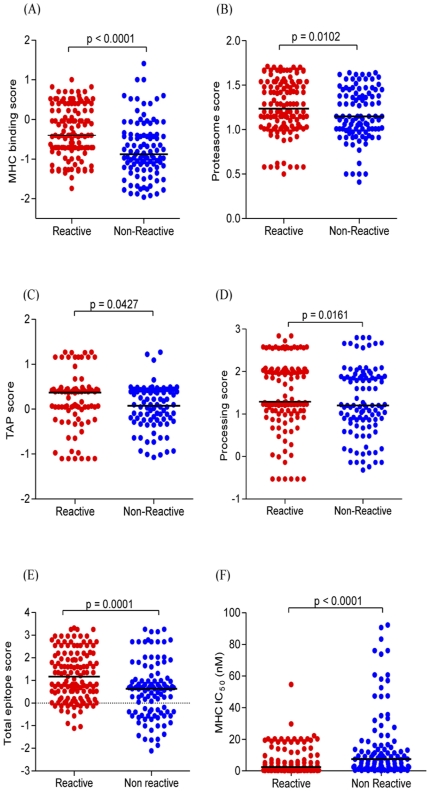
Characterization of peptide processing and MHC class I presentation. Comparison of (A) Major Histocompatibility Complex (MHC) binding score, (B) proteasome cleavage score, (C) transporter associated with antigen processing (TAP) score, (D) processing score, which combines proteasomal cleavage and TAP transport scores, (E) total epitope score, and (F) MHC IC_50_, in nM. The total epitope score is a summary of the proteasomal, TAP and MHC binding scores and between reactive peptide variants (red) and their corresponding non-reactive variants (purple). Peptides binding to HLA class I A and B alleles had their total processing scores and MHC IC_50_ predicted using the Immune Epitope Database (www.immuneepitope.org), and scores were compared using the non-parametric Mann-Whitney test.

To test the hypothesis that the level of peptide recognition was most likely governed by mutations in key residues that are associated with peptide processing and binding to restricting HLA molecules, we applied an algorithm prediction tool (www.immuneepitope.org). The tool assesses predicted scores for Transport Associated with Antigen Processing (TAP) binding (an estimate of the affinity of the peptide with the TAP molecule), MHC binding scores (an estimate of the efficiency of binding to an MHC molecule), proteasome scores (an estimate of cleavage site usage) and processing scores (an estimate of the quantity of peptide present in the endoplasmic reticulum that is available for MHC binding, from a combination of cleavage and transport predictions), and was applied to those participants whose HLA class I A and B alleles were typed in our study (Table S1) and where information on these alleles was available in the database. The total epitope score, which is a summary of the proteasomal, TAP and MHC scores, as well as the MHC IC_50_ of the epitopes restricted by the predicted HLA, are shown in Table S3 (study individual CC23). There were higher scores for proteasome activity, TAP and MHC binding, and lower MHC IC_50_, for peptide variants that were recognized in the IFN-γ ELISPOT assay. Overall, when peptide variants were classified into those that were recognized in the ELISPOT assay and those that were not, the MHC binding score of reactive peptides was significantly higher than that of non-reactive variants (p<0.0001, Figure 5A). In addition, proteasomal cleavage scores (Figure 5B), TAP scores (Figure 5C) and processing scores (Figure 5D) were significantly higher for reactive peptide variants compared to their non-reactive counterparts (p = 0.0102; p = 0.0427 and p = 0.0161, respectively). When all these scores were summated, the total score of the reactive peptides was significantly higher than non-reactive peptide variants (p = 0.0001, Figure 5E). Furthermore, MHC IC_50_ scores of the reactive variants were significantly lower for reactive variants when compared to their corresponding non-reactive variants (p<0.0001, Figure 5F). Thus, non-recognized peptides, with higher entropy, may have at some stage mutated under immune-mediated selection pressure, showing that variability in key residues is important for MHC presentation and recognition by the TCR. Overall, these data provide support for the notion that different clades of HIV-1 may have been shaped by class I HLA restricted epitope diversity through probable selective immune pressures in different populations. However, it cannot be discounted that the non-reactivity of these peptides might be due to random sequence variability, unrelated to immune selection, and that these regions may never be recognized as epitopes.

## Discussion

Immunogens designed to elicit T-cell responses are a major focus of HIV vaccine development. Because of the significant sequence variation that exists between HIV-1 clades (7–15%, [23]), there is an acknowledgement that the HIV clades on which vaccines are based will have an impact on the immune response elicited, and very likely the subsequent efficacy of vaccines.

Testing HIV-infected persons serves as a proxy for assessing T-cell cross-reactivity of highly immunogenic vaccines. It is useful for determining whether HIV-specific responses that particular populations with specific HLA alleles mount, and their degree of cross-reactivity to vaccine sequences that may be tested in that region; infecting viruses represent future ‘challenge’ viruses that vaccine trial volunteers may encounter. Previous studies have shown that HIV-infected individuals can mount cross-reactive T-cell responses against different HIV-1 clades [24]–[27]. While early studies focused on a limited number of selected epitopes, and relied on the use of pools of peptides or cells infected with recombinant vaccinia virus expressing HIV proteins, more recent studies have assessed the degree of cross-clade recognition at the peptide or epitope level [12], [28], [29]. Ours is the first study to comprehensively look at the ability of clade C infected individuals to recognize peptides included in vaccines currently being tested, and to investigate both intra-clade and inter-clade cross recognition.

We investigated T-cell reactivity in 39 individuals where the sequence of the infecting virus was determined by population sequencing of the dominant virus, and high resolution HLA typing was performed in a sub-group of these individuals. Although we did not show directly that responses were due to CD8+ T-cells, reactivity was assumed to be CD8-mediated as previous studies have shown that ELISpot responses are predominantly CD8+ T-cell mediated [30], [31]. However, we cannot rule out the possibility that some of the responses may have been CD4-mediated. In addition, we assessed responses against Gag peptides from five sequence variants, clade C from South Africa based on the Du422 sequence [32], C_CH_ (Chinese clade C, for intra-clade responses), and clades B, A and D, at the single peptide level. Although South Africa and China have genetically distinct clade C epidemics, in this study, neither the magnitude nor the breadth of HIV-specific T-cell responses to Chinese and South African clade C peptide variants differed significantly. The magnitude and breadth of the responses to these two clade C peptide variants was significantly higher than that of Gag peptide reagents based on clades B, A and D sequences. This is further corroboration of results from previous studies which have shown that HIV-specific T-cells are cross-reactive among different HIV clades but with a preference for the infecting clade [14], [15]. A similar reduction in epitope breadth for non-infecting clade peptide sets of approximately 50–70% was observed in a clade B-infected population when comparing recognition of clade B peptides to C and A peptides sets spanning the whole HIV genome [29]. These data suggest that vaccines based on other clades may be cross-reactive and therefore warrant their testing in HIV-1 clade C-epidemic regions. However, whilst vaccines based on non-matching HIV clades may still induce cross-reactive responses, this reactivity may be less than that for clade-matched vaccines. Indeed, Gray et al [16] demonstrated recently in the Phambili phase IIb trial of a clade B-based Ad5 immunogen in a South African population that 12% fewer vaccinees mounted a response to clade C peptides compared to clade B, with a 35% reduction in the overall magnitude of the responses. Whilst the vaccine was not protective in this population, it was also not effective in a clade B-infected population [33], so no conclusions can be drawn regarding the lower clade C-specific responses and vaccine efficacy. Clade-matching vaccines may represent a viable approach for regions where single clades circulate, such as Southern Africa, but this approach is highly limited for regions where multiple clades circulate, and increasing global HIV-1 diversity is a major challenge [34]. Thus, the goal of developing a global vaccine that is as cross-reactive as possible remains.

The results from our study demonstrate that within a single individual, some HIV peptides were exclusively recognized in the clade C sequence variants (C_Du422_ and C_CH_), whilst others were uniquely recognized in the clades B, A and D peptide variant. The recognition of clades B, A and D peptide variants and not the corresponding clade C peptide variants is of importance, as it demonstrates that using a single peptide reagent set leads to a considerable number of responses being missed when investigating T-cell immune responses [35], [36]. These findings are of interest, since a previous study demonstrated no increase in epitope recognition when using centre of tree (COT) and most common recent ancestor (MRCA) peptide sequences in addition to clade B consensus peptides, in a clade B HIV-infected population [37]. Of course, all these approaches very likely provide an underestimation of actual responses, since using peptide reagents matching the autologous virus demonstrates an increase in detectable T-cell responses of 29%, even in more conserved parts of the genome such as p24 [38]. Importantly, a slightly greater increase of 37% in detectable epitopic regions was demonstrated when using a clade B Nef PTE (potential T-cell epitope) peptide set compared to clade B consensus peptides [37]; indeed, preclinical vaccine studies show that these synthetic mosaic immunogens based on the PTE approach expand both the breadth and depth of T-cell responses [39]–[41]. Whether these increases in cross-reactive breadth are sufficient to be cross-protective remains to be elucidated in clinical trials.

Further characterization of reactive peptides in the study identified highly cross-reactive peptides with low intra- and inter-clade diversity, as shown by their lower entropy scores. Peptides that were recognized in two or more variant forms had significantly lower entropy scores when compared to peptides recognized once across the peptide sets. The pattern of recognition observed in the mutually recognized peptides may imply that HLA alleles restricting these peptides are driving mutations in the epitopes, as shown by loss of recognition of certain variants. Further characterization of the mutually recognized peptides identified other key factors including TAP and MHC binding and proteasomal cleavage as playing a role in the recognition of specific variants and not others. This may illustrate the evolution of HIV due to T-cell pressure in HLA class I-restricted epitopes [42]–[47], which is evidenced by HLA footprints observed in specific regions of the viral proteome containing HIV-specific HLA class I restricted T-cell epitopes [48], [49]. This phenomenon of HLA-driven viral evolution was illustrated recently with the first evidence of vaccine-driven T-cell footprints on viral sequences reported in breakthrough infections in the STEP trial [47].

Overall, these data further corroborate previous findings which suggest that within the clades A, B, C and D sequences, some corresponding viral regions share a similar degree of viral diversity, possibly due to structural constraints that prevent sequence mutations in specific parts of the viral genome [50]. This is further supported by the finding that most of the highly cross-recognized peptides were from the p24 region of the Gag protein which is known to be highly conserved and play a structural role in the HIV proteome. Yet, cross-clade recognition of peptides with considerable differences in their amino acid composition was also observed; most of the amino acid changes were semi-conserved, that is between amino acids with closely related side chains, and therefore did not have a significant impact on the processing of the epitopes for presentation by HLA alleles. This suggests that T-cell receptors of HIV-specific T-cells as well as HLA molecules can tolerate some degree of amino acid substitution in their epitopes without total loss of epitope recognition or binding as previously found in other studies [11], [30], [51]. Interestingly, even the same peptide was recognized to different degrees of cross-reactivity in different individuals, showing that different HLA molecules tolerate amino acid changes to different extents, which has to be noted when designing vaccine immunogens to elicit cross-reactive responses in different populations.

In conclusion, this study has shown that clade C HIV-infected individuals recognize peptides based on Chinese and South African sequences equally well, suggesting that intra-clade variability from diverse geographic regions may not necessarily be an impediment to vaccine design. Additionally, while extensive cross-clade recognition was detected, the total magnitude was lower and the breadth of T-cell recognition narrower when compared with intra-clade C T-cell responses. These data suggest that vaccine-induced T-cell immunity of clade-mismatched vaccines would result in lower immunogenicity at the epitope level. A range of approaches are currently being pursued to develop cross-reactive HIV vaccines, including those containing only conserved regions of the HIV proteome among clades [52]–[54], as well as mosaic approaches that seek to represent the majority of the diversity within clades [55]. Immunogens containing conserved regions would serve to focus the T-cell vaccine-induced response towards regions that are less likely to mutate due to structural constraints, and specifically exclude responses to variable regions that may be easily escapable, have little consequence on viral control, and may even act as decoys masking responses to conserved regions [56]. The identification in our study of mutually reactive epitopes within conserved regions of the Gag protein support vaccine design strategies that incorporate conserved regions of the viral genome. Alternatively, T-cell mosaic antigens seek to increase cross-clade reactivity by maximizing the T-cell epitope coverage for most variants [55]. Ultimately, only testing these different vaccine approaches in clinical efficacy trials will inform us of what the best approach is for long-term protection from HIV acquisition or disease.

## Materials and Methods

### Ethics Statement

This study was approved by the ethical review boards of the University of Cape Town and University of the Witwatersrand and each study participant provided written informed consent.

### Participants

Sixty ml of blood was drawn from 40 HIV-1 infected individuals by venipuncture in Acid-Citrate-Dextrose (ACD) tubes. Eligibility criteria were willing and able to provide informed consent, clinically asymptomatic, ART naïve, and with a peripheral blood CD4 count above 350 cells/mm^3^. Peripheral Blood mononuclear Cells (PBMCs) were isolated using standard Ficoll-Hypaque density gradient centrifugation.

### Peptides

A total of 540 peptides were used, 120 each for C_Du422_, C_CH_ and B and 90 peptides each for the A and D sets used. The clade C and B peptides spanned the full length of the Gag protein while clade A and D peptides covered the p17, p24 and p2 regions. Peptides corresponding to p15 were excluded from data comparisons between peptide sets because this region was omitted from the A and D sets. The peptide sets were derived from HIV-1 Gag clades A, D, consensus B, and vaccine insert matched peptides from C_Du422_ clade C (South Africa) and a Chinese clade C strain (C_CH_). Table S2 details preclinical and clinical trials in which vaccines containing the C_Du422_, C_CH_ and A sequences were tested. All peptides sets were provided by the International AIDS Vaccine Initiative, apart from the clade B peptides, which were provided by the National Institute of Health AIDS Research and Reagent Repository.

### HLA typing

High resolution HLA class I A, B and C typing was performed using sequence specific PCR. Briefly, DNA was extracted using the QIAGEN DNA isolation kit for blood (QIAGEN, Chatsworth, CA). High-resolution HLA class I genotyping was performed by sequencing of exons 2, 3 and 4 using Atria Allele SEQR kits (Abbott Diagnostics) and Assign SBT 3.5 (Conexio Genomics, Fremantle, Australia).

### Gag sequencing

HIV RNA was extracted from 140µl of frozen plasma by lysis under highly denaturing conditions followed ethanol washes and elution in 60µl RNAse-free buffer. HIV *gag* cDNA was generated using the Invitrogen Thermoscript^TM^ RT-PCR system (Invitrogen Corp, San Diego, CA). cDNA from the RT step was amplified in a first round PCR using sequence-specific primers, Gag D forward 5”….3′ (HXB2 626-644) and Gag D reverse 5′….3′ (HXB2 2402-2382). Three *gag* regions (A, B and C were amplified separately in a nested PCR using fragment sequence-specific primers, A forward 5”….3”, HXB2 683-704, A reverse 5′….3′, HXB2 1303-1282, B forward, HXB2 1226-1248 and B reverse, HXB2 1846-1825, C forward, 1748-1768 and C reverse, 2356-2334 [57]. The amplified products were bulk sequenced in both 5′ and 3′ directions on an automated ABI 3100 genetic analyser (Applied Biosystems, Inc) in six separate reactions for each study participant. The resulting sequences were assembled using ChromasPro, aligned using BioEdit and phylogenetic analyses performed using MEGA3.

### IFN-γ ELISPOT assay

T-cell responses were assessed by IFN-γ ELISPOT assay as previously described [8]. Briefly, PBMC were plated in 96-well polyvinyledene difluoride-backed plates (Microsep; Millipore Products, France) coated with 250µg of anti-IFN-γ mAb 1-D1K (Mabtech, Sweden) overnight at 4^°^C. The unbound antibody was washed away with three washes with 200µl/well of sterile PBS. Peptides arranged in 5 pools and 24 matrix pools for each of the five peptide sets, were added at a final concentration of 1.5µg/ml. CEF peptide pool (National Institute of Health AIDS Research and Reference Reagent Program) were added at a final concentration of 1.5µg/ml. PBMC were added at 100 000 cells/well and incubated overnight at 5% CO_2_,37^°^C. On the following day, the plates were washed six times with 200µl/well of PBS containing 0.05% Tween 20 (PBS-Tween; Sigma, USA). Biotinylated anti-human IFN-γ monoclonal antibody (7-B6-1, MabTech Sweden), diluted to 2µg/ml in PBS-10% FCS, was added and the plates were incubated for 3 hours at room temperature.

The plates were washed again six times with 200µl/well PBS 0.05% Tween and Streptavidin-Horse-Radish Peroxidase (HRP; BD Pharmingen, Canada) at 1∶500 with PBS-10% FCS was added for one hour at room temperature. The plates were washed for a further six times with 200µl/well of PBS-Tween. The development step was performed with 100µl/well Nova Red substrate (Vector Laboratories, CA) and stopped by rinsing the plate under tap water. Spots were counted on a CTL Analyser (CTL Technologies, Cleveland, USA) and expressed as spot forming units per million (SFU/10^6^) PBMC. A response was considered positive if the count exceeded 100 SFU/10^6^ PBMC after background subtraction. The criteria for a successful assay was less than 5 SFU in each media control well, no more than 10 SFU for background wells (cells only) and greater than 400 SFU in the PHA control wells. Single peptide reactivity was individually confirmed after deconvoluting the pool/matrix reactive peptides in the initial screen. The number of epitopes was determined by taking into consideration overlapping peptides. Two consecutive peptides were considered as one response and three consecutive peptides as two responses.

### Peptide binding affinity and epitope processing prediction

Binding affinities in the legend of Table 2 were predicted using NetMHC3.0 [58], [59]. The effect of amino acid mutations on the different steps involved in epitope processing and presentation by their respective HLA alleles was investigated by predicting proteasomal cleavage scores, transport by transport associated with antigen processing (TAP) and MHC class I binding scores [60]–[62] using a computational method (www.immuneepitope.org). The analysis was performed on all peptides reactive in the study and the proteasome, TAP, MHC; processing and total scores were predicted as well as the MHC IC_50_.

### Statistical analysis

Statistical analysis was performed using GraphPad version 5.00 for Windows (Prism Software, San Diego, California USA). All data were analysed by use of non-parametric statistics. The Friedman one way ANOVA test for matched pairs was performed to test for any significant differences in genetic distances, magnitude and breadth of responses among the different peptide set. This was followed by a Dunn's post-test for multiple comparisons in the case of any significant Friedman p value. All analyses involving comparison amongst the different peptide sets are based on p17p24p2 region of the HIV Gag protein. The Kruskal-Wallis test was performed to test for differences in the median entropies of peptides in different recognition categories. The Mann-Whitney test for unmatched pairs was used to test for significant differences in the total scores and MHC IC_50_ values between reactive and non-reactive peptide variants. All tests were two-tailed and p values of<0.05 were considered significant.

## Supporting Information

Figure S1 **Characterization of peptides recognized in the study.** (A) Number of individuals responding to each peptide. (B) Number of peptide variants recognized for each reactive peptide. (C) Average entropy score of the five peptide variants for each peptide recognized. The Gag region from which each reactive peptide is located is shown at the bottom of the figure.
(TIF)

Table S1 **Clinical characteristics of study participants.**
(DOC)

Table S2 **Gag peptide sets and sequences.**
(DOC)

Table S3 **The effect of amino acid mutations on predictions of epitope processing.**
(DOC)
