# Supplementary figures and images for: Intra- and Inter-clade Cross-reactivity by HIV-1 Gag Specific T-Cells Reveals Exclusive and Commonly Targeted Regions: Implications for Current Vaccine Trials
Source: PLoS One. 2011 Oct 12;6(10):e26096. doi: 10.1371/journal.pone.0026096 (PMC3192159; doi:10.1371/journal.pone.0026096)

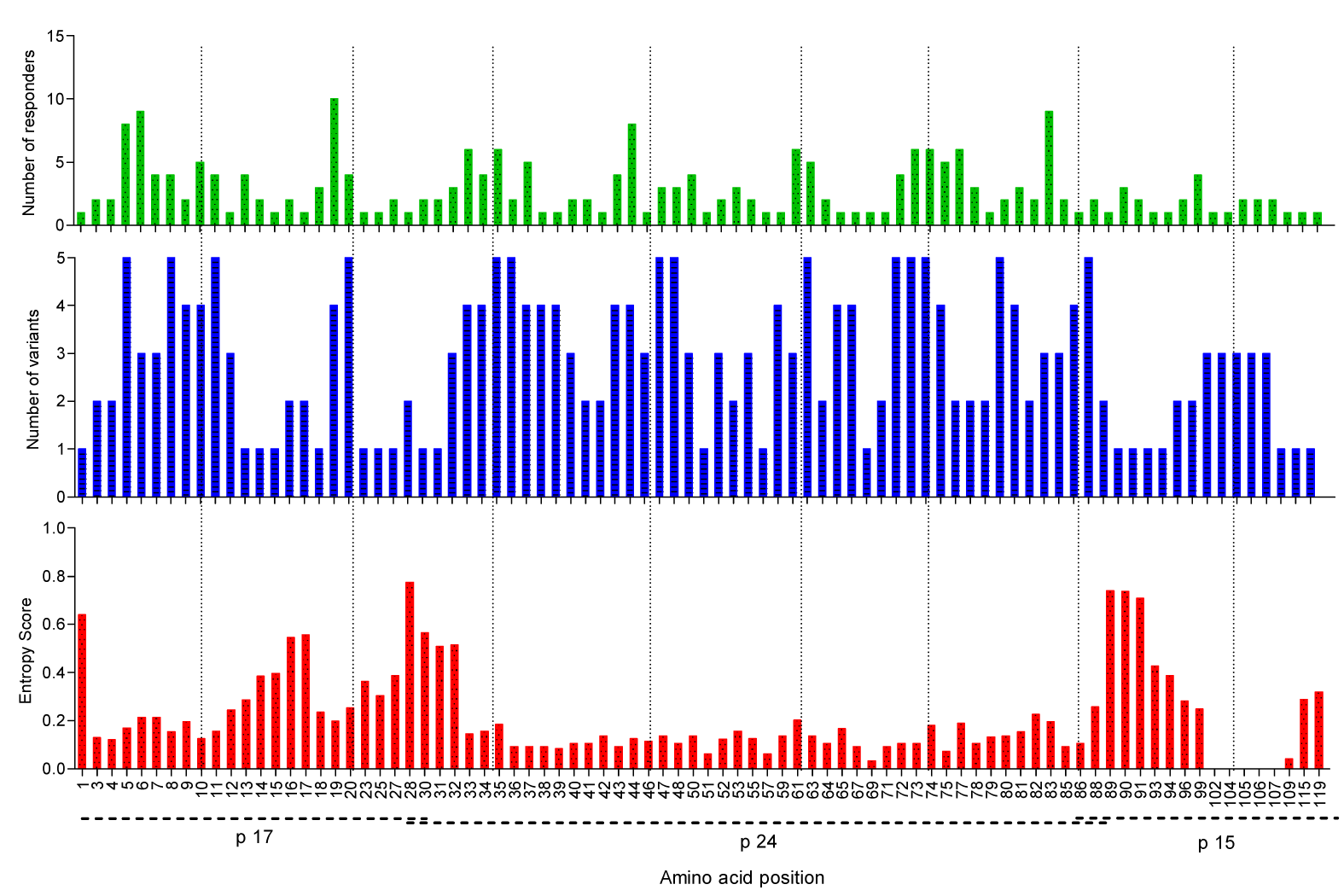

Supplement: Figure S1 — Characterization of peptides recognized in the study. (A) Number of individuals responding to each peptide. (B) Number of peptide variants recognized for each reactive peptide. (C) Average entropy score of the five peptide variants for each peptide recognized. The Gag region from which each reactive peptide is located is shown at the bottom of the figure. (TIF) [file pone.0026096.s001.tif]
