# Supplementary material for: Intra- and Inter-clade Cross-reactivity by HIV-1 Gag Specific T-Cells Reveals Exclusive and Commonly Targeted Regions: Implications for Current Vaccine Trials
Source: PLoS One. 2011 Oct 12;6(10):e26096. doi: 10.1371/journal.pone.0026096 (PMC3192159; doi:10.1371/journal.pone.0026096)
Supplement: Table S1 — Clinical characteristics of study participants. (DOC) [file pone.0026096.s002.doc]

**Table S1. Clinical characteristics of study participants**

| **P.I.D** | **Age**  **(years)** | **CD4 count**  **(cells/µl)** | **Viral load**  **(copies/ml)** | **HLA-A** | | **HLA-B** | | **HLA-C** | |
| --- | --- | --- | --- | --- | --- | --- | --- | --- | --- |
| **CC05** | 32 | 610 | 3900 | A2902 | A3002 | B4201 | B4201 | C1701 | C1701 |
| **CC12** | 47 | 536 | 11000 | A 3001 | A3002 | B070201 | B5801 | C0302 | C0702 |
| **CC15** | 27 | 452 | 180000 | A 0201 | A2902 | B080101 | B4201 | C1701 | C1701 |
| **CC17** | 40 | 884 | 43000 | A 0205 | A2601 | B5101 | B1401 | n/a | C1804 |
| **CC19** | 23 | 569 | 7800 | A 3002 | A7401; A7402 | B070201 | B1503; B9503 | C0202; C0210 | C0702 |
| **CC22** | 31 | 420 | 15000 | A 2301 | A3303 | B0801; B0803 | B5301; B3529 | n/a | n/a |
| **CC23** | 31 | 565 | 72000 | A0201 | A3002 | B080101 | B4501 | n/a | C1601 |
| **ST03** | 29 | 578 | 21000 | A3001 | A7401; A7402 | B4201 | B5801 | C0302 | C1701 |
| **NM06** | 27 | 1321 | 130000 | A3002 | A4301 | B1510 | B570301 | C0401; C0409N | C1801; C1802 |
| **NM07** | 35 | 1010 | 18000 | A0101 | A3001 | B1503; B9503 | B4501 | C0401; C0409N | C0602 |
| **BN08** | 28 | 1437 | 740 | A0101 | A3001 | B1503; B9503 | B4501 | C0401; C0409N | 0602 |
| **MT09** | 33 | 605 | 2700 | A2301;A2304 | A2902; A2903 | B1503; B9503 | B4201 | C0210 | C1701 |
| **RL12** | 19 | 628 | 3500 | A2301; A2301 | A290201;A2903 | B510101 | B1401 | C0702 | C0727 |
| **TM16** | 29 | 702 | 15000 | A2301 | A2301 | B0801 | B4501 | C0602; C0611 | C0304; C0307 |
| **ER17** | 35 | 999 | 2000 | A2901 | A3404 | B1801; B1817N | B8101; 8102 | C0704; C0711 | C0804 |
| **HN18** | 29 | 471 | 11000 | A2902 | A7401 | B1503;B9503 | B5802 | C0210; C0202 | C0602 |
| **NS19** | 34 | 1167 | 3200 | A3001 | A7401; A7402 | B4201 | B5802 | C1701 | C0302 |
|  |  |  |  |  |  |  |  |  |  |

N = 17 had complete HLA typing for loci A and B. N = 2 had no complete HLA typing for C locus. Suffix N denotes HLA alleles whose protein is known not to be expressed and n/a denotes data not available.
