# Supplementary material for: Intra- and Inter-clade Cross-reactivity by HIV-1 Gag Specific T-Cells Reveals Exclusive and Commonly Targeted Regions: Implications for Current Vaccine Trials
Source: PLoS One. 2011 Oct 12;6(10):e26096. doi: 10.1371/journal.pone.0026096 (PMC3192159; doi:10.1371/journal.pone.0026096)
Supplement: Table S2 — Gag peptide sets and sequences. (DOC) [file pone.0026096.s003.doc]

**Table S2. Gag peptide sets and sequences**

| **Peptide set name** | **HIV sequence information and references** | **Vaccine trials and references** |
| --- | --- | --- |
| **Clade A** | Gag A p24/p17 consensus; [1]Hanke *et al*., 2000 | [2]Peters *et al*., 2007; [3]Jaoko *et al*., 2008 |
| **Clade B** | Gag B Consensus; Catalogue # 8116 NIH AIDS Research Reference Reagent Program [https://www.aidsreagent.org](https://www.aidsreagent.org/) | Not Applicable |
| **South Africa clade C (CDu422)** | C_Du422_ [4]Williamson *et al*., 2003; GenBank: AF544010 | [5]Burgers *et al*., 2009 |
| **China clade C (CCH)** | C/B’ synthetic; [6]Huang *et al*., 2008 | [7-9]Vasan *et al*., 2010a, b, 2011 |
| **Clade D** | Gag D p24/p17 consensus; Courtesy of Bette Korber, LANL, Los Alamos, NM | Not Applicable |
|  |  |  |

**References**

1. Hanke T, McMichael AJ (2000) Design and construction of an experimental HIV-1 vaccine for a year-2000 clinical trial in kenya. Nat Med 6(9): 951-955.

2. Peters BS, Jaoko W, Vardas E, Panayotakopoulos G, Fast P, et al (2007) Studies of a prophylactic HIV-1 vaccine candidate based on modified vaccinia virus ankara (MVA) with and without DNA priming: Effects of dosage and route on safety and immunogenicity. Vaccine 25(11): 2120-2127.

3. Jaoko W, Nakwagala FN, Anzala O, Manyonyi GO, Birungi J, et al (2008) Safety and immunogenicity of recombinant low-dosage HIV-1 A vaccine candidates vectored by plasmid pTHr DNA or modified vaccinia virus ankara (MVA) in humans in east africa. Vaccine 26(22): 2788-2795.

4. Williamson C, Morris L, Maughan MF, Ping LH, Dryga SA, et al (2003) Characterization and selection of HIV-1 subtype C isolates for use in vaccine development. AIDS Res Hum Retroviruses 19(2): 133-144.

5. Burgers WA, Chege GK, Muller TL, van Harmelen JH, Khoury G, et al (2009) Broad, high-magnitude and multifunctional CD4+ and CD8+ T-cell responses elicited by a DNA and modified vaccinia ankara vaccine containing human immunodeficiency virus type 1 subtype C genes in baboons. J Gen Virol 90(Pt 2): 468-480.

6. Huang Y, Chen Z, Zhang W, Gurner D, Song Y, et al (2008) Design, construction, and characterization of a dual-promoter multigenic DNA vaccine directed against an HIV-1 subtype C/B' recombinant. J Acquir Immune Defic Syndr 47(4): 403-411.

7. Vasan S, Hurley A, Schlesinger SJ, Hannaman D, Gardiner DF, et al (2011) In vivo electroporation enhances the immunogenicity of an HIV-1 DNA vaccine candidate in healthy volunteers. PLoS One 6(5): e19252.

8. Vasan S, Schlesinger SJ, Chen Z, Hurley A, Lombardo A, et al (2010a) Phase 1 safety and immunogenicity evaluation of ADMVA, a multigenic, modified vaccinia ankara-HIV-1 B'/C candidate vaccine. PLoS One 5(1): e8816.

9. Vasan S, Schlesinger SJ, Huang Y, Hurley A, Lombardo A, et al (2010b) Phase 1 safety and immunogenicity evaluation of ADVAX, a multigenic, DNA-based clade C/B' HIV-1 candidate vaccine. PLoS One 5(1): e8617.
