# Supplementary material for: Intra- and Inter-clade Cross-reactivity by HIV-1 Gag Specific T-Cells Reveals Exclusive and Commonly Targeted Regions: Implications for Current Vaccine Trials
Source: PLoS One. 2011 Oct 12;6(10):e26096. doi: 10.1371/journal.pone.0026096 (PMC3192159; doi:10.1371/journal.pone.0026096)
Supplement: Table S3 — The effect of amino acid mutations on predictions of epitope processing. (DOC) [file pone.0026096.s004.doc]

Table S3. The effect of amino acid mutation on predictions of epitope processing

| **Peptide number** | **Peptide variant** | **Peptide**  **sequence** | **SFU/10^6^ PBMC** | **Proteasome score** | **TAP score** | **MHC binding score** | **Processing score** | **Total score** | **MHC IC_50_** |
| --- | --- | --- | --- | --- | --- | --- | --- | --- | --- |
| **7** | C_Du422_ | -KKHYMLKHIVWASRE | 1750 | 1.1 | -0.23 | 0.7 | 0.87 | 1.57 | 0.20 |
|  | C_CH_ | -........L...... | 940 | 1.1 | -0.23 | 0.7 | 0.86 | 1.56 | 0.20 |
|  | B | G..K.K......... | 0 | 1.1 | -0.24 | -1.12 | 0.86 | -0.25 | 13.1 |
|  | A | -..K.R...L...... | 0 | 1.1 | -0.19 | -1.21 | 0.90 | -0.30 | 16.1 |
|  | D | -..K.R...L...... | 0 | 1.1 | -0.19 | -1.21 | 0.90 | -0.30 | 16.1 |
| **11** | C_Du422_ | -ERFALNPGLLETSEG | 130 | 0.97 | -0.29 | -0.59 | 0.67 | 0.08 | 3.9 |
|  | C_CH_ | -............... | 130 | 0.97 | -0.29 | -0.59 | 0.67 | 0.08 | 3.9 |
|  | B | L....V......... | 0 | 0.97 | -0.27 | -1.09 | 0.70 | -0.39 | 12.3 |
|  | A | -.......S....A.. | 0 | 0.96 | -0.29 | -0.63 | 0.67 | 0.04 | 4.3 |
|  | D | -............... | 130 | 0.97 | -0.29 | -0.59 | 0.67 | 0.08 | 3.9 |
| **15** | C_Du422_ | -KQIMKQLQPALQTGT | 0 | 0.84 | -0.35 | -1.35 | 0.48 | -0.87 | 22.4 |
|  | C_CH_ | -...I........... | 0 | 0.84 | -0.35 | -1.35 | 0.48 | -0.87 | 22.4 |
|  | B | CR..LG....S.... | 120 | 1.01 | -0.65 | -1.47 | 0.36 | -1.12 | 29.6 |
|  | A | CQ...E...S..K.SE | 0 | 0.87 | -0.73 | -1.52 | 0.14 | -1.38 | 33.1 |
|  | D | -...IG.....I...S | 0 | 0.90 | -1.02 | -1.50 | -0.13 | -1.63 | 31.9 |
| **19** | C_Du422_ | -ELKSLYNTVATLYCV- | 0 | 1.15 | 0.05 | -0.38 | 1.20 | 0.82 | 2.40 |
|  | C_CH_ | -..R..F......... | 1280 | 1.15 | 0.07 | 0.52 | 1.22 | 1.75 | 0.30 |
|  | B | E..R........... | 520 | 1.15 | 0.07 | -0.40 | 1.22 | 0.83 | 2.50 |
|  | A | --....F.........H | 2353 | 1.15 | 0.05 | 0.40 | 1.20 | 1.60 | 0.40 |
|  | D | -..R............ | 480 | 1.15 | 0.07 | -0.40 | 1.22 | 0.83 | 2.50 |
| **38** | C_Du422_ | -AWVKVIEEKAFSPEV | 470 | 1.03 | 0.05 | 0.52 | 1.08 | 1.61 | 0.30 |
|  | C_CH_ | -............... | 470 | 1.03 | 0.05 | 0.52 | 1.08 | 1.61 | 0.30 |
|  | B | N.....V........ | 0 | 0.41 | -0.73 | -0.88 | -0.32 | -1.20 | 7.50 |
|  | A | -............... | 470 | 1.03 | 0.05 | 0.52 | 1.08 | 1.61 | 0.30 |
|  | D | -............... | 470 | 1.03 | 0.05 | 0.52 | 1.08 | 1.61 | 0.30 |
| **63** | C_Du422_ | -PPIPVGDIYKRWIIL | 6670 | 1.54 | 0.45 | -0.44 | 1.99 | 1.95 | 1.10 |
|  | C_CH_ | -..V...E........ | 5980 | 1.54 | 0.44 | 0.22 | 1.98 | 2.20 | 0.60 |
|  | B | N......E....... | 5320 | 1.54 | 0.44 | 0.22 | 1.98 | 2.20 | 0.60 |
|  | A | -............... | 6670 | 1.54 | 0.45 | -0.44 | 1.99 | 1.95 | 1.10 |
|  | D | -......E........ | 5520 | 1.54 | 0.44 | 0.22 | 1.98 | 2.20 | 0.60 |
| **80** | C_Du422_ | -LVQNANPDCKTILRA | 7470 | 1.67 | 0.39 | -0.71 | 2.06 | 1.35 | 5.20 |
|  | C_CH_ | -............... | 7470 | 1.67 | 0.39 | -0.71 | 2.06 | 1.35 | 5.20 |
|  | B | L.............K | 3920 | 1.67 | 0.39 | -0.71 | 2.06 | 1.35 | 5.20 |
|  | A | -..........S.... | 2653 | 1.17 | 0.25 | -1.26 | 1.42 | 0.16 | 18.4 |
|  | D | -.............K. | 1240 | 1.67 | 0.39 | -0.71 | 2.06 | 1.35 | 5.20 |
| **89** | C_Du422_ | -HKARVLAEAMSQTNS | 110 | 0.94 | -0.94 | -0.22 | -0.01 | 0.22 | 0.60 |
|  | C_CH_ | -............A.G | 0 | 0.77 | -0.54 | 0.12 | 0.22 | 0.35 | 0.80 |
|  | B | G............VT | 0 | 1.05 | 0.32 | 1.41 | 0.72 | 0.68 | 25.6 |
|  | A | -......GTGARASVL | 0 | 1.52 | 0.33 | -1.76 | 1.85 | 0.08 | 58.0 |
|  | D | -............ATN | 200 | 0.98 | -0.50 | 0.15 | 0.47 | 0.63 | 0.70 |
| **107** | C_Du422_ | FLGKIWPSHKGRPGN | 450 | 0.58 | -1.10 | 0.46 | -0.53 | -0.13 | 0.40 |
|  | C_CH_ | ............... | 450 | 0.58 | -1.10 | 0.46 | -0.53 | -0.13 | 0.40 |
|  | B | ............... | 450 | 0.58 | -1.10 | 0.46 | -0.53 | -0.13 | 0.40 |

The different stages of epitope processing were predicted using algorithms for MHC class I T-cell epitope processing for reactive peptides in the study. Results shown in the table are for individual CC23. For peptide 107, the A and D variants are missing due to the absence of the p15 region in these peptide reagent sets, as noted in the text.
